# Healthy migrants but unhealthy offspring? A retrospective cohort study among Italians in Switzerland

**DOI:** 10.1186/1471-2458-12-1104

**Published:** 2012-12-22

**Authors:** Silvan Tarnutzer, Matthias Bopp

**Affiliations:** 1 Institute of Social and Preventive Medicine, University of Zurich, Hirschengraben 84, 8001, Zurich, Switzerland

## Abstract

**Background:**

In many countries, migrants from Italy form a substantial, well-defined group with distinct lifestyle and dietary habits. There is, however, hardly any information about all-cause mortality patterns among Italian migrants and their offspring. In this paper, we compare Italian migrants, their offspring and Swiss nationals.

**Methods:**

We compared age-specific and age-standardized mortality rates and hazard ratios (adjusted for education, marital status, language region and period) for Swiss and Italian nationals registered in the Swiss National Cohort (SNC), living in the German- or French-speaking part of Switzerland and falling into the age range 40–89 during the observation period 1990–2008. Overall, 3,175,288 native Swiss (48% male) and 224,372 individuals with an Italian migration background (57% male) accumulated 698,779 deaths and 44,836,189 person-years. Individuals with Italian background were categorized by nationality, country of birth and language.

**Results:**

First-generation Italians had lower mortality risks than native Swiss (reference group), but second-generation Italians demonstrated higher mortality risks. Among first-generation Italians, predominantly Italian-speaking men and women had hazard ratios (HRs) of 0.89 (95% CI: 0.88-0.91) and 0.90 (0.87-0.92), respectively, while men and women having adopted the regional language had HRs of 0.93 (0.88-0.98) and 0.96 (0.88-1.04), respectively. Among second-generation Italians, the respective HRs were 1.16 (1.03-1.31), 1.06 (0.89-1.26), 1.10 (1.05-1.16) and 0.97 (0.89-1.05). The mortality advantage of first-generation Italians decreased with age.

**Conclusions:**

The mortality risks of first- and second-generation Italians vary substantially. The healthy migrant effect and health disadvantage among second-generation Italians show characteristic age/sex patterns. Future investigation of health behavior and cause-specific mortality is needed to better understand different mortality risks. Such insights will facilitate adequate prevention and health promotion efforts.

## Background

Migrants to European countries (e.g., Sweden, Belgium and the Netherlands) were reported to have lower overall mortality rates than the respective local population [1-3]. A similar difference has been documented for Latino immigrants to the United States [4,5]. In Europe as well as in the U.S., migrants are generally characterized by low socio-economic status (SES) and working in unhealthy jobs. Low SES has been shown to be related to unfavorable health behavior, opportunities and outcomes (e.g., physical inactivity, smoking, unhealthy dietary habits, difficulties in accessing health services, morbidity and mortality) [6-8]. This phenomenon of lower mortality in the socio-economically disadvantaged Latino population in the U.S. was called the “Latino mortality paradox” [9]. This term was later modified in the European context to “Mediterranean migrants’ mortality paradox” [10].

Established theoretical explanations focus on selective migration flows of healthy workers (healthy-migrant hypothesis). Others assume that protective social and cultural factors from the country of origin entail general health advantages,e.g., lower suicide rates among migrants from Southern Europe [11,12]. Generally, the impact of the culture of origin diminishes with increasing length of stay [12]. The literature suggests that beliefs and norms concerning health behavior change due to acculturation [13]. Language is a proxy for acculturation. Furthermore, it has repeatedly been shown that language is an important predictor of health care utilization and health status [14]. However, it has been reported that acculturation processes and SES are interlinked and should both be considered [14].

Methodical and conceptual considerations are related to the choice of the reference population (host population vs. non migrating cohort in the country of origin) and distinguish between ‘arrival’ and ‘post-arrival’ phase’ [15]. Further difficulties refer to the re-migration of sick individuals (salmon-bias hypothesis) [3,9] and under-reported re-emigration (‘over-coverage’ [16]), bothbiasing mortality figures. However, several findings suggest that the lower mortality among migrants is at least partially real [3,5,9,17]. With the Swiss National Cohort (see Methods, below), we substantially reduce the over-coverage and other numerator/denominator problems occurring in mortality analyses among migrant populations.

There is abundant literature investigating cancer among Italian migrants, e.g. [18]. However, to our knowledge systematic analyses on all-cause mortality are lacking. This paper presents differences in all-cause mortality between Swiss nationals and individuals with an Italian background. Italians were the most numerous foreign population group in Switzerland since World War II, reaching a maximum of 555,000 persons in 1974 (seasonal workers not included) - i.e., 52% of all foreign nationals or almost 9% of the overall population of Switzerland at that time [19].

In Switzerland, health care insurance is mandatory, and access to health care is regarded to be universal [20]. However, while lower overall hospitalization rates were reported for migrants generally, Italians had higher rates for admissions due to ischemic heart diseases [21]. Self-reported health of immigrants has also been lower than that of Swiss nationals [22].

In our analyses, we investigate the hypotheses that compared to native Swiss (1) Italian immigrants possess an all-cause mortality advantage whereas (2) their offspring demonstrate higher all-cause mortality risk, but differences in mortality are (3) moderated by the adoption of Swiss regional languages.

## Methods

### Data

Data were derived from the Swiss National Cohort (SNC), an anonymous record linkage of census, cause of death and emigration files [23]. Ethics approval (Nr. 13/06) was obtained by the Ethics Committee of the Canton of Zurich (Kantonale Ethik-Kommision, KEK).

Between 1850 and 2000, national censuses were conducted in Switzerland at 10-year intervals in early December of a census year and information about sex, age, country of birth, nationality, educational level and marital status is taken from the 1990 census. Marital status was categorized into single, married, widowed and divorced/separated. Educational level was classified according to the International Standard Classification of Education (ISCED): compulsory schooling (ISCED levels 0–2), secondary (3), higher secondary (4) and tertiary education (5–6) [24]. Usual minimal numbers of years for each educational category in Switzerland are 9, 11–12, 14–15 and 16, respectively. Swiss census enumeration and registration of deaths occurring in Switzerland (including cause of death information) is presumed to be virtually complete [25].

In multilingual persons, the preferred language is also a significant marker of cultural identity. A special feature of Swiss censuses is the assessment of mastered languages. Until 1980, censuses included only one language item (“What language do you think in and do you master best?”), permitting only a single response. Although in 1990 this question was complemented by additional language questions, the quality of these items is unsure. Hence we restricted the analyses to the more reliable question concerning principal language. One out of four Italians born in Switzerland still reported Italian as their main language. Nevertheless, it can be assumed that the vast majority of individuals with Italian background can understand both Italian and the regional language, and that the principal language indicated stands more for mental acculturation than actual fluency.

For comparisons with the non-migrating cohort in the country of origin, cross-sectional mortality and population data for Italy was derived from the WHO mortality database (http://www.who.int/whosis/mort/download/en/).

### Migration background

In this study, migration background is conceptualized by considering both citizenship (addressed as nationality below) and birthplace. In Swiss law, citizenship is acquired through descent (jus sanguinis) or naturalization (after a relatively long stay in the country and sufficient knowledge of regional language).

Migration status was categorized into four groups:

1) First-generation Italians (in analyses referred to as “IT, *IT”): Italian nationality, born in Italy

2) Second-generation Italians (IT, *CH): Italian nationality, born in Switzerland

3) Italy-born Swiss (CH, *IT): Swiss nationality, born in Italy

4) Native Swiss (CH, *CH): Swiss nationality, born in Switzerland

The category “Italy-born Swiss” encompasses both naturalized Italian immigrants and native Swiss born in Italy. In the 1990 census, only one nationality could be reported. Swiss nationals with additional citizenship were instructed to declare themselves as “Swiss”. Therefore, we have no information on individuals’ possible second nationalities. For multivariate analyses, we further differentiated people with Italian background into those who reported Italian as principal language and those reporting the region-specific language (German or French).

### Inclusion/exclusion criteria

The current SNC database encompasses 6,873,687 persons registered in the 1990 census and 1,037,335 deaths occurring between the 1990 census and the end of 2008. Individuals with non-Swiss and non-Italian nationality or born in neither Switzerland nor Italy were excluded. We further excluded persons living in the Italian-speaking part of Switzerland (~4%) or reporting a language other than Italian or the regional Swiss language. Cut-off points for age at baseline were set at 25 and 90 years. However, individuals contributed person years and deaths only after their 40th birthday. The high lower age limit is due to the reliance on educational data from the 1990 census, implying that generally even the youngest individuals included in our analyses should have reached their highest educational level by 1990. We further excluded 186,732 individuals which could neither be linked to a 2000 census record nor were documented to have died or emigrated.

Each individual added person-years starting at the 40th birthday and ending either one day before the 90th birthday, date of death or emigration or December 31, 2008. With respect to all mentioned conditions, 3,399,660 individuals accumulating 698,779 deaths and 44,836,189 person years were included.

Observation time was partitioned into two periods. The first one begins on the day of the 1990 census (December 4, 1990) and ends on the 2000 census day (December 5, 2000). Within this time-span, follow-up status of the whole study population is known. The second period starts at December 5, 2000 and ends on December 31, 2008 and contains an unknown number of individuals lost to follow-up (all persons without linked death or emigration record have to be assumed to be alive at the end of 2008).

### Procedures

Statistical analyses were done in Stata 11.2 [26] and cover descriptive statistics (counts, means and relative frequencies), age-standardized (WHO standard population “Europe”) mortality rates and multivariate models (Cox regressions). The proportional hazard assumption appeared to be widely fulfilled.

## Results

### Descriptive analyses

**Figure 1:**
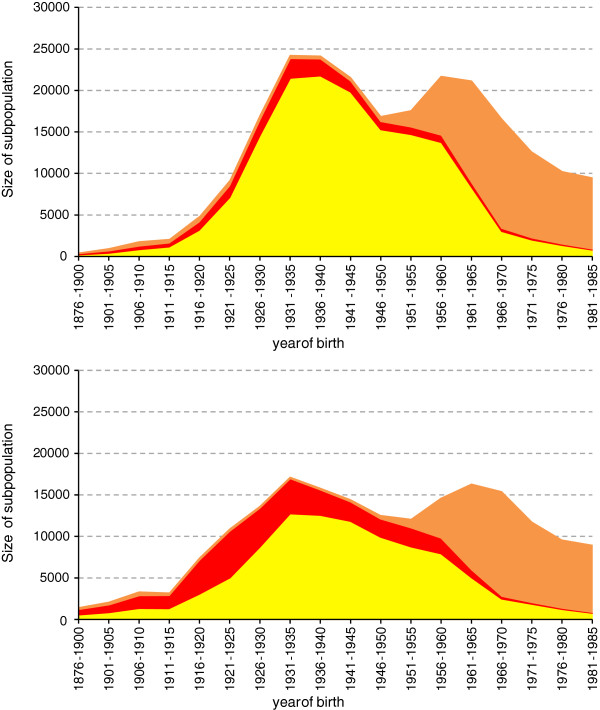
**Age-specific size of subpopulation 1990 among men (top) and women (bottom).** Yellow area: Italian nationals, born in Italy; red area: Swiss nationals, born in Italy; orange area: Italian nationals, born in Switzerland. Data source: Swiss Federal Statistical Office/Swiss National Cohort.

In 1990 and depending on age class, male and female Italian nationals made up to 11% and 7%, respectively, of Switzerland’s population (Figure 1). Furthermore, second-generation Italians accounted for up to 6% of the population in the birth cohorts 1966–1990.

Among Italian nationals – irrespective of place of birth – men outnumbered women slightly, whereas almost three out of four Italy-born Swiss nationals were women (Table 1). Generally, educational achievement was higher in Swiss than in Italian nationals and in men than in women. Among both men and women, Swiss nationals born in Italy had the highest proportion of tertiary education. Variation in marital status between the four migration categories broadly reflects differences in age structure. For men and women, proportions of Italian nationals were higher in French- than in German-speaking Switzerland.

**Table 1:** Study population, Swiss National Cohort, 1990–2008, 25–89 years at baseline

		**Men**				**Women**				
		IT, *IT	IT, *CH	CH, *IT	CH, *CH	IT, *IT	IT, *CH	CH, *IT	CH, *CH	**Total**
Individuals included in analysis	German-speaking Switzerland	74,528	8,315	5,250	1,192,789	46,231	5,482	14,110	1,293,754	2,640,459
	French-speaking Switzerland	32,068	3,807	2,983	323,026	20,529	2,400	8,669	365,719	759,201
	German- + French-speaking Switzerland	106,596	12,122	8,233	1,515,815	66,760	7,882	22,779	1,659,473	3,399,660
Person-years	1990-2000	748,976	34,473	68,081	10,631,070	480,393	19,316	190,449	11,935,429	24,108,187
	2000-2008	589,674	64,436	51,598	9,200,327	383,932	41,987	146,106	10,249,943	20,728,002
Deaths	1990-2000	6,241	1,166	1,132	213,898	2,658	414	2,480	180,515	408,504
	2000-2008	6,323	607	885	148,670	2,746	252	2,286	128,506	290,275
Mean age on 1990,12,4		46.0	38.2	53.0	49.4	47.0	36.5	55.9	51.4	50.2
Education (%)	Compulsory schooling	59.1	21.4	26.9	19.6	81.7	33.7	62.4	39.5	32.2
	Secondary	35.5	64.7	49.8	57.7	16.8	62.4	32.6	54.5	54.5
	Upper Secondary	3.9	11.0	13.2	15.1	0.8	2.8	2.4	3.9	8.9
	Tertiary	1.4	3.0	10.2	7.5	0.7	1.1	2.6	2.1	4.5
Marital status (%)	Single	11.1	41.6	8.1	19.2	7.7	41.8	3.4	15.8	17.1
	Married	83.4	51.5	83.4	72.2	81.9	50.1	71.6	63.0	68.1
	Widowed	1.3	2.5	3.1	3.5	7.1	4.6	16.9	14.5	9.0
	Separated/divorced	4.3	4.5	5.5	5.1	3.4	3.5	8.2	6.7	5.9
Main language (%)	Italian	88.6	23.1	43.7	3.1	91.3	28.4	49.2	3.8	8.4
	Preponderant regional language	11.4	77.0	56.4	96.9	8.7	71.6	50.8	96.2	91.6

Data source: Swiss Federal Statistical Office/Swiss National Cohort.

### Age-standardized mortality rates

In general, age-standardized mortality rates were lower in first-generation Italians than native Swiss (Table 2). However, the difference was only significant among women. In contrast, second-generation migrants had higher mortality rates than native Swiss. Finally, Italy-born Swiss demonstrated lower mortality than native Swiss.

**Table 2:** Age-standardized mortality rates per 100,000 person-years, 1990–2008 individuals aged 25–89 years at baseline

**Migration category**	**Men**	**(n: 1,642,766)**		**Women**	**(n:1,756,894)**	
	**Deaths(n)**	**Mortality rates**	**95%-CI**	**Deaths(n)**	**Mortality rates**	**95%-CI**
IT, *IT	12564	1532	1493-1572	5403	820	796-845
IT, *CH	1773	1944	1843-2045	666	1024	924-1124
CH, *IT	2015	1373	1310-1436	4765	847	820-875
CH, *CH	362542	1550	1545-1555	308981	884	880-887

Data source: Swiss Federal Statistical Office/Swiss National Cohort.

CI = confidence interval.

* = born in; example: IT, *IT: Italians born in Italy i.e. first-generation Italians.

**Figure 2:**
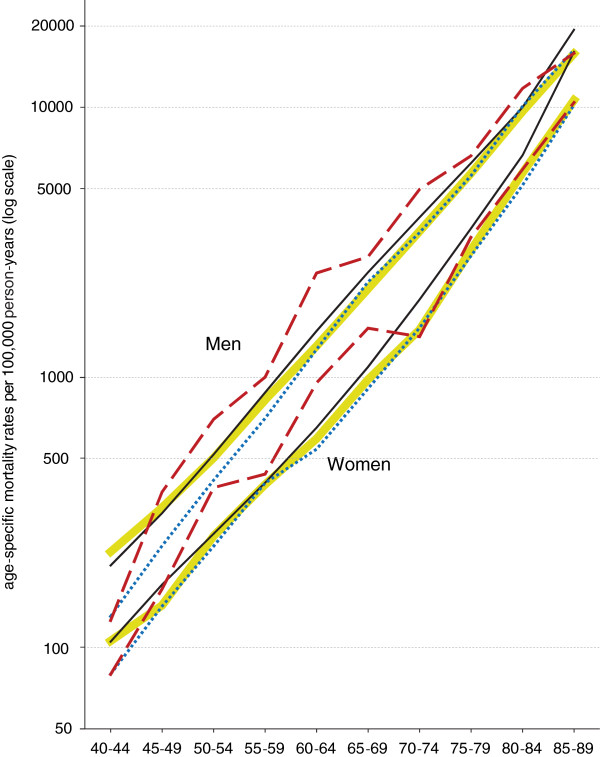
**Age-specific mortality rates 1991–2000 among men (top) and women (bottom).** Dotted blue lines: Switzerland, first-generation Italians; dashed red lines: Switzerland, second-generation Italians; yellow thick lines: native Swiss nationals; black thin lines: population of Italy. Data sources: Swiss Federal Statistical Office/Swiss National Cohort and WHO mortality database (Italy).

For an evaluation of variation by age, we compared age-specific rates (averaged over the years 1991–2000) with information from the WHO Mortality Database for Italy. The comparison showed nearly identical rates for Italy’s population and native Swiss nationals – only Swiss aged over 60 years had a mortality advantage (Figure 2). The healthy migrant effect of first-generation Italian migrants was clearly observable for men younger than 60 years and women younger than 50 years. Second-generation Italians aged less than 45 years had lower and those aged between 45 and 70 years had higher mortality compared to Italy’s population as well as native Swiss.

### Multivariate analyses

Among men, Cox regressions with the explanatory variables migration group (including best mastered language), linguistic region (German vs. French), age, marital status, educational level and a period dummy showed significantly different mortality risks for all migration categories compared to native Swiss (Table 3). The lowest risks were observed in first-generation Italian-speaking Italians (HR: 0.89; CI: 0.88-0.91). First-generation Italians who adopted the regional language also demonstrated a lower risk (HR: 0.93; CI: 0.88-0.98), as well as Italy-born Swiss (irrespective of language). In contrast, Switzerland-born Italians had increased mortality risk (Italian speakers: HR = 1.16, CI: 1.03-1.31; regional language speakers: HR = 1.10, 1.05-1.16).

**Table 3:** Cox regression 1990–2008, individuals aged, 25–89 years at baseline.

		**Men**	**(n: 1,642,766)**	**Women**	**(n: 1,756,894)**
		**HR**	**95%-CI**	**HR**	**95%-CI**
Age	Age	**1.02**	1.02-1.02	**1.01**	1.01-1.02
Migrational group	IT, *IT, Italian	**0.89**	0.88-0.91	**0.90**	0.87-0.92
	IT, *IT, Regional	**0.93**	0.88-0.98	0.96	0.88-1.04
	IT, *CH, Italian	**1.16**	1.03-1.31	1.06	0.89-1.26
	IT, *CH, Regional	**1.10**	1.05-1.16	0.97	0.89-1.05
	CH, *IT, Italian	**0.91**	0.86-0.98	**0.94**	0.90-0.98
	CH, *IT, Regional	**0.94**	0.88-0.99	0.99	0.95-1.03
	CH, *CH (ref.)	1		1	
Educational level	Compulsory school (ref.)	1		1	
	Secondary education	**0.84**	0.83-0.85	**0.83**	0.83-0.84
	Higher secondary education	**0.69**	0.68-0.69	**0.73**	0.71-0.75
	Tertiary education	**0.59**	0.58-0.60	**0.68**	0.65-0.71
Marital status	Single	**1.49**	1.48-1.51	**1.29**	1.28-1.31
	Married (ref.)	1		1	
	Widowed	**1.18**	1.17-1.20	**1.16**	1.15-1.17
	Separated/divorced	**1.55**	1.53-1.57	**1.36**	1.34-1.38
Language region	German-speaking Switzerland (ref.)	1		1	
	French-speaking Switzerland	1.00	1.00-1.01	**0.95**	0.94-0.96
Period	1990-2000 (ref.)	1		1	
	2000-2008	**0.94**	0.92-0.95	**0.94**	0.93-0.96

Data source: Swiss Federal Statistical Office/Swiss National Cohort, HR = hazard ratio; CI = confidence interval figures in bold: significant on 5%-level.

* = born in; example: CH, *IT, Italian: Italy born Swiss who see Italian as their best mastered language.

In women, two migrant groups had significantly different mortality risk compared to native Swiss, but only when adhering to Italian as main language (Table 3): first-generation Italians and Italy-born Swiss had HR’s of 0.90 (CI: 0.87-0.92) and 0.94 (0.90-0.98), respectively. No effect was observed for Switzerland-born Italians and for any group having adopted the regional language.

The findings also showed the higher the educational level, the lower the mortality risk. People living alone showed significantly higher mortality compared to married people. Mortality was lower in French- than German-speaking Switzerland among women, but not among men. Additional models including interaction terms between migration background and language region or period showed no remarkable differences.

## Discussion

First-generation Italians had a mortality advantage compared to native Swiss. This is in line with international findings-e.g., a Swedish study on migrants from southern Europe, ex-Yugoslavia and Turkey [1] and the only Swiss study on mortality among foreigners [27] which reported lower age-standardized mortality rates for foreigners compared to Swiss nationals (men −19%, women −14%). However, the latter study was cross-sectional and did not distinguish between migrants and their offspring. Nevertheless, the mortality advantage of Italian immigrants in Switzerland may be a real phenomenon rather than an artifact. This notion is also supported by a Belgian study based on register data [3].

We also found that second-generation Italian men were exposed to higher mortality risk. In this group, adoption of the regional language attenuated the excess mortality observed. Other authors have also documented higher mortality rates in the offspring of migrants. In England and Wales, second- and third-generation Irish people aged 15–44 years demonstrated excess mortality [28,29]. Sons of Italian migrants in Sweden had a higher mortality risk than native Swedish nationals [12]. This is consistent with the acculturation hypothesis. As the duration of stay increases, immigrants increasingly adopt the local lifestyle and protective features of their culture of origin are only partially maintained by their offspring [12]. But for mortality, the acculturation hypothesis has been confirmed in some, but not all migrant populations [30-32].

In our study, the mortality advantage in first-generation Italians was greater for men than women but diminished in men and lost significance in women who had adopted the regional language.

In a study on second-generation Italians in Switzerland, two main types of orientation were differentiated: “casual Latins” emphasized their Italian origin and only rarely got involved in Switzerland’s political agenda and discourses [33]. A similar observation was made for Italians living in Peterborough, UK, characterized as economically integrated but socially encapsulated [34]. In contrast to the “casual Latins” the “sushi-eating secondos” adhered to a cosmopolitan kind of Italian culture and lifestyle and signalized interest in cultural diversity and Swiss values [33]. It may be assumed that adoption of the regional language corresponds with these constructed ideal types. If adoption of the regional language is interpreted as an indicator of acculturation, our results are also in line with the attenuation of health advantages over time as described above.

Notwithstanding the larger proportion of people with an Italian migration background in the French-speaking part of Switzerland, separate Cox regression models could not discern clear differences in mortality risk between Italians in German- vs. French-speaking Switzerland, irrespective of adoption of the regional language.

Controlling for period revealed a period effect which may be caused by undetected loss to follow-up in the second period. Apart from this, the hazard ratios were very similar when calculated for both periods separately. The relationships between migration background and mortality may therefore be assumed to be stable over time.

### Strengths and limitations

The mortality advantage of first-generation Italians in the context of our study is meaningful as we avoid the numerator-denominator bias inherent to cross-sectional studies by using SNC data. Individuals were selected if they had taken part in both Swiss censuses (1990 and 2000) or are documented to have died or emigrated. This criterion is important because many migrants who re-emigrate do not report their move to the authorities. Indeed, in Sweden, it was estimated that 10% of the immigrants who leave the country do not fulfill this obligation in order to keep an option open for re-migration or access to better health care than in their country of origin [1,35]. In France, the proportion of missing deaths among Moroccan men was estimated at 23% [10]. However, the SNC also includes deaths which had occurred abroad as covered by the foreigners’ registration system, but not the routine mortality statistics. In the first period, this applies to 381 deaths (4% of deaths of first-generation and 1% of Switzerland-born Italians), in the second period to 933 (10% and 4.5%, respectively).

A few methodical limitations should be mentioned. First, for the second period (after the 2000 census), information was missing for an unknown number of individuals having emigrated without notification and some deaths not linkable to the SNC. Restricting the analyses to the first period (1990–2000) may enhance reliability but at the expense of statistical power. Sensitivity analyses showed essentially the same patterns when limited to data from the first period. We, therefore, preferred to control the pooled analyses with a period dummy. Second, we had no information on the exact origin of migrants. Since there is a substantial south–north gradient in mortality within Italy [36,37], analyses with this additional information may help explain differences in mortality between different migration groups. Third, the migration category ‘Swiss nationals born in Italy’ does not permit distinction between naturalized Italian immigrants and the offspring of Swiss nationals born in Italy. A fourth limitation is the lack of data on the duration of stay and on immigration class. The majority of Italians in Switzerland immigrated during the 1950s and 1960s. It is, therefore, possible that the observed differences between migration categories are confounded by cohort effects. However, immigration class should not bias our results.

## Conclusions

We found significant differences in mortality between native Swiss and specific groups of individuals with Italian background in Switzerland. We observed substantial variation between first- and second-generation Italians: while first-generation males and females had a significant survival advantage compared to Swiss nationals, second-generation males demonstrated a significantly higher mortality risk. Given the substantial number of second-generation Italians, their poorer health might become a significant public health burden in the future. The situation of migrants and their offspring with respect to poorer self-reported and actual health status [2,12,21,22,38] calls for public health actions focused on second-generation Italians. Further investigations should, therefore, take a closer look at potential resources (e.g., social networks, dietary patterns) and specific risks in health behavior, morbidity and cause-specific death rates, in order to customize preventive and health promotion efforts.

## Competing interests

The authors declare that they have no competing interests.

## Authors’ contributions

MB conceived the study, prepared the data and contributed to the interpretation and writing of the manuscript. ST performed the analyses, contributed to the interpretation of the results, and wrote the first draft of the manuscript. Both authors read and approved the final version of the manuscript.

## Pre-publication history

The pre-publication history for this paper can be accessed here:

http://www.biomedcentral.com/1471-2458/12/1104/prepub

## References

[B1] Weitoft GR Gullberg A Hjern A Rosén M Mortality statistics in immigrant research: method for adjusting underestimation of mortality Int J Epidemiol 1999 28 756 763 10.1093/ije/28.4.756 10480707

[B2] Bos V Kunst AE Keij-Deerenberg IM Garssen J Mackenbach JP Ethnic inequalities in age- and cause-specific mortality in The Netherlands Int J Epidemiol 2004 33 1112 1119 10.1093/ije/dyh189 15166193

[B3] Deboosere P Gadeyne S Adult migrant mortality advantage in Belgium: evidence using census and register data Population - English edition 2005 60 655 698

[B4] Singh GK Hiatt RA Trends and disparities in socioeconomic and behavioural characteristics, life expectancy, and cause-specific mortality of native-born and foreign-born populations in the United States, 1979–2003 Int J Epidemiol 2006 35 903 919 10.1093/ije/dyl089 16709619

[B5] Singh GK Siahpush M All-cause and cause-specific mortality of immigrants and native born in the United States Am J Public Health 2001 91 392 399 11236403 10.2105/ajph.91.3.392 PMC1446566

[B6] Adler NE Boyce T Chesney MA Cohen S Folkman S Kahn RL Syme SL Socioeconomic status and health: the challenge of the gradient Am Psychol 1994 49 15 24 8122813 10.1037//0003-066x.49.1.15

[B7] Marmot MG Shipley MJ Rose G Inequalities in death—specific explanations of a general pattern? Lancet 1984 323 1003 1006 10.1016/S0140-6736(84)92337-7 6143919

[B8] Marmot MG Smith GD Stansfeld S Patel C North F Head J White I Brunner E Feeney A Health inequalities among British civil servants: the Whitehall II study Lancet 1991 337 1387 1393 10.1016/0140-6736(91)93068-K 1674771

[B9] Abraído-Lanza AF Dohrenwend BP Ng-Mak DS Turner JB The Latino mortality paradox: a test of the "salmon bias" and healthy migrant hypotheses Am J Public Health 1999 89 1543 1548 10.2105/AJPH.89.10.1543 10511837 PMC1508801

[B10] Khlat M Darmon N Is there a Mediterranean migrants mortality paradox in Europe? Int J Epidemiol 2003 32 1115 1118 10.1093/ije/dyg308 14681289

[B11] Johansson LM Sundquist J Johansson SE Bergman B Qvist J Träskman-Bendz L Suicide among foreign-born minorities and Native Swedes: an epidemiological follow-up study of a defined population Soc Sci Med 1997 44 181 187 10.1016/S0277-9536(96)00142-6 9015871

[B12] Sundquist K Li X Coronary heart disease risks in first- and second-generation immigrants in Sweden: a follow-up study J Intern Med 2006 259 418 427 10.1111/j.1365-2796.2006.01630.x 16594910

[B13] Abraído-Lanza AF Armbrister AN Flórez KR Aguirre AN Toward a theory-driven model of acculturation in public health research Am J Public Health 2006 96 1342 1346 10.2105/AJPH.2005.064980 16809597 PMC1522104

[B14] Carter-Pokras O Bethune L Defining and measuring acculturation: a systematic review of public health studies with hispanic populations in the united states. A commentary on Thomson and Hoffman-Goetz Soc Sci Med 2009 69 992 995 10.1016/j.socscimed.2009.06.042 19631433

[B15] Gushulak BD MacPherson DW The basic principles of migration health: population mobility and gaps in disease prevalence Emerg Themes Epidemiol 2006 3 3 10.1186/1742-7622-3-3 16674820 PMC1513225

[B16] Gadd M Johansson SE Sundquist J Wändell P Are there differences in all-cause and coronary heart disease mortality between immigrants in Sweden and in their country of birth? A follow-up study of total populations BMC Public Health 2006 6 102 10.1186/1471-2458-6-102 16630338 PMC1475577

[B17] Marmot MG Adelstein AM Bulusu L Immigrant mortality in England and Wales, 1970–78: Causes of death by country of birth 1984 H.M.S.O, London

[B18] Geddes M Parkin DM Khlat M Balzi D Buiatti E Cancer in Italian migrant populations 1993 Lyon: International Agency for Research on Cancer IARC scientific publications, vol 123 8365772

[B19] Swiss Federal Statistical Office Statistisches Jahrbuch der Schweiz 1979 1979 Basel: Birkhäuser Verlag

[B20] Bilger V Hollomey C Wyssmüller C Efionayi-Mäder D Health Care for undocumented Migrants in Switzerland: Policies - People - Practices 2011 Vienna: International Center for migration policy development

[B21] Moreau-Gruet F Luyet S Migrationsbevölkerung und Gesundheit – Analyse der Hospitalisierungen 2012 Neuchâtel: Swiss Health Observatory Obsan-Bulletin, vol 1

[B22] Bischoff A Wanner PH The self-reported health of immigrant groups in Switzerland J Immigrant Minority Health 2008 10 325 335 10.1007/s10903-007-9089-z 17939053

[B23] Bopp M Spoerri A Zwahlen M Gutzwiller F Paccaud F Braun-Fahrländer C Rougemont A Egger M Cohort profile: the Swiss National Cohort-a longitudinal study of 6.8 million people Int J Epidemiol 2008 38 379 384 10.1093/ije/dyn042 18326512

[B24] United Nations Educational, Scientific and Cultural Organisation: International standard classification of education: ISCED 1997 1997 Paris: Unesco

[B25] Polasek W Schuler M Eidgenössische Volkszählung 1990 1996 Bern: Swiss Federal Statistical Office

[B26] StataCorp LPstata11: Stata Statistical Software 2009 TX: StataCorp., College Station

[B27] Wanner PH Bouchardy C Raymond L Mortalité des étrangers en Suisse: Analyse par grand groupe des causes et par type de cancer 1989–1992 2000 Neuchâtel: Swiss Federal Statistical Office

[B28] Harding S Balarajan R Patterns of mortality in second generation Irish living in England and Wales: longitudinal study BMJ 1996 312 1389 1392 10.1136/bmj.312.7043.1389 8646095 PMC2351124

[B29] Adelstein AM Marmot MG Dean G Bradshaw JS Comparison of mortality of Irish immigrants in England and Wales with that of Irish and British nationals Ir Med J 1986 79 185 189 3744754

[B30] Singh GK Miller BA Health, life expectancy, and mortality patterns among immigrant populations in the United States Can J Public Health 2004 95 I14 21 15191127 10.1007/BF03403660 PMC6975697

[B31] Harding S Mortality of migrants from the Indian subcontinent to England and Wales: effect of duration of residence Epidemiology 2003 14 287 292 12859028

[B32] Harding S Mortality of migrants from the Caribbean to England and Wales: effect of duration of residence Int J Epidemiol 2004 33 382 386 10.1093/ije/dyh059 15082645

[B33] Wessendorf S Sushi-eating secondos and casual Latins: political movements and the emergence of a latino counter-culture among second-generation Italians in Switzerland J Intercult Stud 2007 28 345 360 10.1080/07256860701429758

[B34] Tubito M King R Italians in Peterborough: between integration, encapsulation and return 1996 Falmer: Geography Laboratory, University of Sussex

[B35] Westerling R Rosén M 'Avoidable' mortality among immigrants in Sweden Eur J Public Health 2002 12 279 286 10.1093/eurpub/12.4.279 12506503

[B36] Fascioli S Capocaccia R Mariotti S Cancer mortality in migrant populations within Italy Int J Epidemiol 1995 24 8 18 10.1093/ije/24.1.8 7797360

[B37] Rasulo D Spadea T Onorati R Costa G The impact of migration in all-cause mortality: the Turin longitudinal study, 1971–2005 Soc Sci Med 2012 74 897 906 10.1016/j.socscimed.2011.10.045 22326305

[B38] Carballo M Divino JJ Zeric D Migration and health in the European Union Trop Med Int Health 1998 3 936 944 10.1046/j.1365-3156.1998.00337.x 9892278

